# Study on forecasting method of power engineering cost based on BIM and DynGCN

**DOI:** 10.1371/journal.pone.0322202

**Published:** 2025-05-08

**Authors:** Huijing Zhai, Jiangtao Ma

**Affiliations:** Shandong Electric Power Engineering Consulting Institute Corp., Ltd., Jinan, China; Dr NGP Institute of Technology (A Unit of Kovai Medical Center and Hospital), INDIA

## Abstract

In view of the shortcomings of power engineering cost in precision and dynamic in big data environments, this paper proposes building information modelling (BIM) and spatiotemporal modelling-based dynamic graph convolutional neural networks (DynGCN). This study uses the characteristics of BIM technology to carry out the cost management of the whole life cycle of power engineering, and realizes the dynamic control of the cost. In addition, the DynGCN method is used to predict the cost of each engineering link, so as to optimize the construction scheme of the whole project. The results show that the whole life cycle data management supported by BIM technology improves the real-time monitoring and adjustment ability of the cost; the DynGCN method can greatly improve the accuracy of the cost prediction, and the prediction accuracy is 96%, which is closest to the real value of the cost.

## 1 Introduction

Engineering cost is not only a key component of engineering design, but also an important factor affecting the overall implementation of the project. In the power engineering project, the reasonable optimization of cost management can not only ensure the construction order and quality of the project, but also effectively reduce the project cost [1,2]. In recent years, building information modelling (BIM) technology has been widely used because of its comprehensive, accurate and dynamic characteristics. BIM technology is able to provide detailed and real-time information during the design, construction, and operational phases by creating and managing digital 3D models of engineering projects. Its application makes the engineering design more accurate, the construction process more efficient, and can dynamically track the project progress and cost changes [3].

Combined with BIM technology, engineering cost can not only greatly improve the quality of project development and construction, but also identify potential design problems at an early stage. It also provides real-time updates on engineering progress and cost data, thus helping the engineering team to move forward with project implementation faster and better, and to make necessary adjustments and optimizations [3]. Huan proposed a complete BIM attribute information inspection method for the information interaction of project participants, and constructed an engineering cost prediction model to guide the project decision-making. But the model does not involve the cost of each stage of the engineering [4]. Qi uses BIM modelling and Unity3D technology to quickly mine the massive value data in the project to improve the benefit of engineering decision-making. But the research focuses on data visualization, and the application effect in cost prediction is yet to be demonstrated [5]. Ding used the Mann-Kendall trend test method and the three exponential smoothing methods (MK-TESM) to build an engineering cost prediction model with high reliability. But the cost control of the whole life cycle of the project needs to be improved [6].

Although BIM technology has been widely used in many fields, its application in power engineering cost management is still relatively small. Especially in the context of the Internet of Things and big data, the existing BIM technology is still slightly insufficient in the comprehensive analysis of the data mining results of various parties and accurate prediction. The current BIM technology has not been able to fully solve the problem of data support in the implementation of the engineering, which may not provide sufficient accuracy in practical applications. This technical deficiency limits the full application of BIM technology in the field of power engineering cost; thus, further research and development is needed to enhance its capability in data integration and prediction accuracy [7].

Traditional methods for engineering cost prediction also have some shortcomings. Ning et al. used 3D convolutional neural networks (3DCNN) for engineering cost estimation [8]. The results of the study conclude that 3DCNN shows excellent performance in the regression problem of cost estimation and gains high application value. However, CNN models require a lot of computational resources and time for training when dealing with engineering cost prediction. And CNN is usually good at processing image data, and may not be as effective as other models specialized for structured data for structured power engineering cost data. Dong et al. proposed a new framework based on long and short-term memory neural networks (LSTM) to explore the applicability of the algorithm and optimization mechanism in the field of cost metrics prediction [9]. The results show that the proposed LSTM framework has a significant advantage in prediction due to its ability to handle high-dimensional feature vectors and to selectively record historical information. Compared with methods such as support vector machines (SVM), the framework has the advantages of being able to capture long-distance dependency information and provide short-term forecasts of engineering cost indicators effectively and accurately. However, LSTM models are relatively complex with many parameters and a long training process, which may lead to overfitting or difficulty in adjusting hyperparameters. Wang et al. proposed a construction cost prediction model based on recurrent neural networks. It was concluded that when the RNN model predicts the engineering cost, the model output has a smaller root mean square error and has a more desirable prediction accuracy [10]. However, RNN models have limitations in capturing long-term dependencies and cannot effectively deal with the complex temporal sequences or dependencies involved in power engineering cost. The ANN-based power project cost prediction model is proposed based on the machine learning mechanism of an artificial neural network (ANN), where historical data samples are normalized as inputs, the network is trained by the ANN algorithm, and the trained network is used to estimate the project cost [11]. However, the ANN algorithm has limited data processing capabilities and does not perform well with complex structured data; moreover, its generalization ability is limited, making it less effective in handling non-specific types of data. A power project cost prediction model based on improved SVM, by considering the components of power project cost and normalizing the parameters. Improvement of SVM model using least squares estimation; meanwhile, genetic algorithm is used to solve the optimal values of parameters of LSSVM, and the optimized GA-LSSVM model is used to achieve the prediction of power engineering cost [12]. However, the improved SVM model may not be able to learn and adapt in real-time to changes during the project process, which is a disadvantage for predicting the ever-changing costs of engineering projects. The random forest (RF) method was used for data mining to eliminate redundant indicators. And Wolf Pack algorithm (WPA) was used to optimize SVM to solve the problems of SVM overfitting and local optimality, so as to improve the accuracy and stability of power engineering cost prediction [13]. However, the method of optimizing SVM parameters using the RF-WPA hybrid algorithm has drawbacks such as insufficient utilization of data features, lack of spatial and temporal dynamic modelling, poor interpretability, and high requirements for data quality. Doing approximate compression of historical data using the approximate theory of properties of rough sets. Combining Monte Carlo simulation algorithms to predict the cost per unit of capacity to the total project cost for sub-projects with certain probability distributions. [14]. However, the Monte Carlo algorithm relies on a large number of simulations to estimate results, which can lead to a complex model with high computational costs. Moreover, the Monte Carlo algorithm may be too dependent on specific simulation parameters and assumptions, limiting its generalization ability across different projects and scenarios.

In order to solve the shortcomings of the above methods in engineering cost prediction, this study proposes a power engineering cost optimization algorithm based on BIM and dynamic graph convolutional neural networks (DynGCN) based on spatio-temporal modelling in a big data environment. The algorithm utilizes DynGCN to predict the engineering cost of each segment on the basis of realizing the whole life cycle management of electric power projects using BIM technology. The method can handle both spatial and temporal dependencies and is suitable for dealing with the complex spatio-temporal data relationships involved in power engineering. And DynGCN can dynamically adjust the graph structure, which makes the model more flexible to adapt to the dynamic changes and different dependencies in the power engineering cost data [15].

## 2 Whole life cycle cost management of electric power engineering

**Fig 1:**
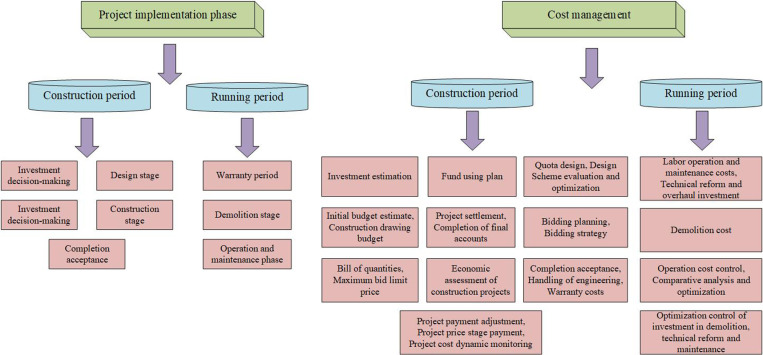
Power engineering whole life cycle cost management content.

BIM is a web-based information-based building design technology for planning, designing, constructing, and managing buildings and infrastructures whose constituent elements include owners, manufacturers, developers, construction managers, contractors, and others. If a change is made to one of the design objects, it will be reflected in every element designed using that object, so users can work collaboratively to continually update the model. This characteristic provides ideas for constructing the whole life cycle management model of the project, recording all the data of the project through BIM technology, optimizing the engineering and technical solutions, and adjusting the parameters in time [16]. This not only improves the quality of the engineering, but also saves the engineering cost and realizes the corresponding design objectives. At the same time, with the further development of grid intelligence, the difficulty of power engineering projects is also rising, for their project cost control has become more complicated. There are many types and unclear division of labor in the cost control of electric power engineering. In addition, the project planning guidelines are difficult to control, and the price estimation of new technology building materials is vague. This leads to the waste of building materials and increased construction costs [17]. In addition, the phenomenon of frequent engineering changes during the construction of power projects, resulting in the formulation of the budget has a large error. Therefore, in response to the above situation, a full life cycle cost management model applicable to electric power engineering is designed using BIM technology. In which the content of full life cycle cost management of electric power engineering is shown in Fig 1.

BIM technology is characterized by coordination, visualization, simulation, support for image output and high cost accuracy [18]. Therefore, in the process of engineering construction control, data security can be guaranteed by virtue of BIM’s data hierarchical categorization system and automatic accumulation system, which facilitates the management and analysis of data. At the same time, according to the needs of each department, the coordination of BIM technology is utilized to complete the data profiling in order to improve the efficiency of all departmental collaboration. In addition, BIM technology can show in detail the use of costs at each stage of the engineering and estimate the progress and cost of the engineering using simulation to effectively control the construction progress and costs. The whole life cycle cost management model of an electric power project constructed based on BIM technology not only improves the accuracy of the construction budget, but also supports prefabrication processing. This substantially improves the level of construction management.

## 3 Cost optimization algorithm for power projects

Based on the BIM technology platform, it is possible to construct visualization models for various engineering schemes, and each scheme has a corresponding cost estimation model. Thus, by linking the BIM model to the cost estimate, the estimated cost of each scenario is generated. Then, by virtue of the engineering implementation simulation and program improvement functions of BIM technology, each candidate construction program is comprehensively compared and analyzed. Thus, it can deepen the pre-project planning, further improve the studyability of the project, and provide effective support for the construction decision-making of the project [19].

**Fig 2:**
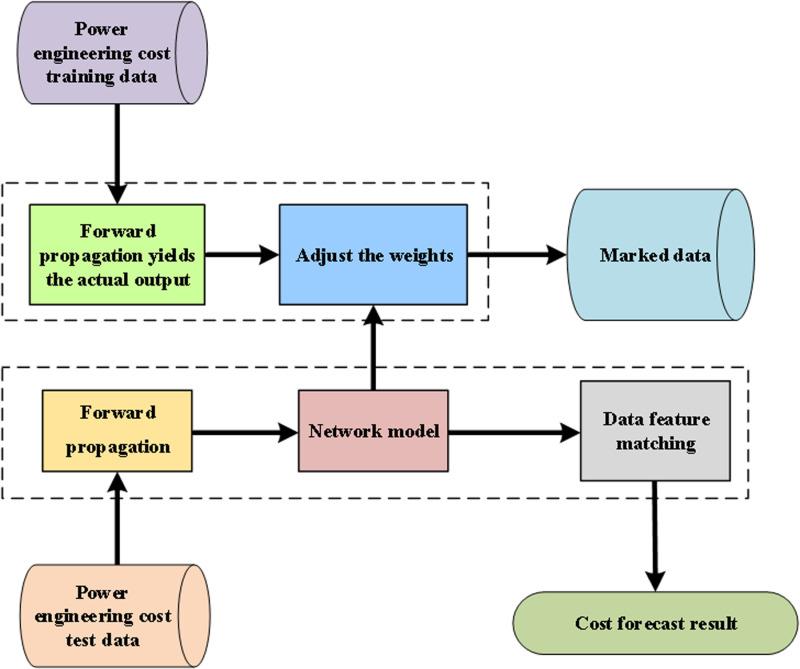
DynGCN-based cost prediction process for power engineering.

The cost of power engineering includes the costs incurred at various stages throughout its life cycle. In this study, the data set is obtained with the help of BIM technology, and DynGCN is utilized for cost prediction, and the prediction process is shown in Fig 2.

Firstly, the trained power engineering data are simply normalized to complete the pre-processing operations such as data denoising and normalization. In order to avoid the interference of possible bad data in the training data on the network training, the batch mode is utilized for network training. That is, a certain number of training samples are arbitrarily chosen to form a small sample as an input. After calculating the iterations until the iteration stop condition is satisfied, the network training is completed. Then the experimental data of the cost of electric power engineering is inputted into the trained DynGCN network, and finally the predicted optimized value of the cost is obtained.

DynGCN, as one of the deep neural network models, is now widely used in the field of data profiling. Its weight-sharing network architecture is similar to biological neural networks, which can simplify the network model and shrink the amount of weights [20–22]. DynGCN is introduced as follows:

### 3.1 Dynamic graphical representation of learning methods

Define dynamic graphs and representation learning on dynamic graphs as follows: a dynamic graph is represented as a sequence $G=\;\;\;\{\;\;\;{\text{G}}_{1}\text{,}{\text{G}}_{2}\text{,}...\text{,}{\text{G}}_{T}\;\;\;\}\;\;\;$ of multiple static graphs, where $G_{t}=(V_{t},E_{t})$ denotes a snapshot at the moment t, t∈{1,2,...,T}. For an adjacency matrix $A_{t}\in R^{N\times N}$, of a graph $G_{t}$, if ${\left(v_{t}\right)}_{i}$ and ${\left(v_{t}\right)}_{j}$ are connected by an edge, then ${\left(A_{t}\right)}_{i,j}\text{=1}$, otherwise ${\left(A_{t}\right)}_{i,j}\text{=0}$. A node on a dynamic graph represents learning as a sequence of mappings $F=\{f_{1},f_{2},\ldots ,f_{T}\},\forall t\in \{1,2,\ldots ,T\}$ where each mapping maps the node at moment t to a low-dimensional vector ${\left(y_{t}\right)}_{v}=f_{t}(\nu )$, such that the mapped vector preserves the original information of the node [23]. This means that if two points are more similar in the original graph, the closer their mapped vectors are.

### 3.2 Model architecture

**Fig 3:**
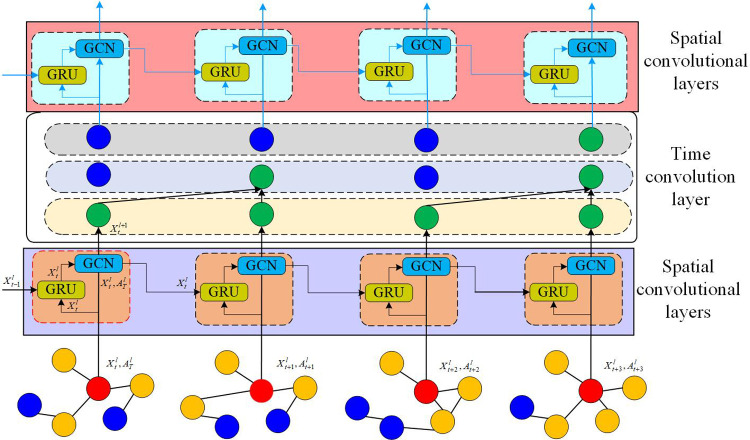
DynGCN model architecture.

The model proposed in this paper models the learning of representation on dynamic graphs as the aggregation of temporal and spatial information, and at the same time joins the model adaptive mechanism, which can update the model parameters along with the changes of the graph structure. As shown in Fig 3, the basic architecture of the model consists of spatial convolutional layers and temporal convolutional layers, and the first layer of the model consists of a spatial convolutional layer to aggregate the node’s neighbour information, and at the same time, the parameters are adaptively updated by using the gated recurrent unit (GRU) unit [24]. The output vectors are fed into the second temporal convolutional layer, which aggregates the information of the current and historical moments. As a result, each node’s representation incorporates both current and historical information about its neighbors. Finally, an adaptive spatial convolutional layer is added to aggregate the current and historical information of the neighbours.

In Fig 3, the red dot represents the central node in the spatial aggregation, the yellow dot represents the first-order neighbor, the green dot represents the data flow direction in the historical information aggregation, and the blue dot represents the inactive node in the spatio-temporal aggregation.

### 3.3 Spatial convolutional layers

In this study, the graph structure extraction advantage of graph neural networks (GNN) is utilized to learn the structural information under each time slice [25]. GCN extends the idea of convolution to graphs by aggregating neighbor information through defined spectrogram convolution. Formally, for the graph at time *t*, the inputs to the lth layer of the GCN are the vectorand the adjacency matrixoutput from the *l*-1th layer, and the output is the updated node vector. The operation of layer *l* is expressed as:


$$\begin{array}{c} X_{t}^{l+1}=\text{F}(X_{t}^{l},A_{\text{t}},W_{t}^{l})=\sigma ({\hat{D}}_{t}^{-1/2}{\hat{A}}_{t}{\hat{D}}_{t}^{-1/2}X_{t}^{l}W_{t}^{l}) \end{array}$$ (1)

where the superscript *l* represents the *l*th graph convolution layer, and the subscript *t* denotes the tth time step; ${\hat{A}}_{t}=A_{\text{t}}+I$; ${\hat{D}}_{t}=\text{diag}(\sum_{j=1}^{N}{\hat{A}}_{t_{\left(ij\right)}})$; I are the unit matrices; operation ${\hat{D}}_{t}^{-1/2}{\hat{A}}_{t}{\hat{D}}_{t}^{-1/2}$ is a normalization of the adjacency matrix D to serve as an approximate graph convolution filter; E is the weight matrix of the *l*th layer at moment *t*; and the function *σ* is the activation function (ReLU). The input $X_{t}^{0}$ of the first layer of the network is *t*he feature matrix of the nodes at time t. Each row of the matrix is a K-dimensional feature vector of each node. After L layers of graph convolutiona*l* layers, the neighbor information of nodes is aggregated in the output vector of each time slice.

Considering the dynamic nature of the graph, the dynamic graph convolutional layer adds an update mechanism to the static GCN architecture. Because when the graph structure changes, the weight parameters of the convolution operation should also be updated to adapt to the new graph structure. The dynamic graph convolutional layer uses the recurrent neural network (RNN) component to update the weight parameters of the GCN model, and for each t∈{1,2,...,T} and l∈{1,2,...,L}, the RNN component takes the initial value of the parameter $W_{t}^{l}$ as the input, and outputs the updated $W_{t}^{l}$. The dynamic graph convolutional layer uses the initial value of the parameter $W_{t}^{l}$ as the input, and outputs the updated $W_{t}^{l}$. The dynamic graph convolutional layer uses the initial value of the parameter $W_{t}^{l}$ as the output. Although multiple implementations of RNNs can serve this purpose, our architecture adopts the GRU implementation since the inputs to the GRU structure contain node representations at each moment, which can introduce richer information about the graph structure for updating the weight parameters.


$$\begin{array}{c} W_{t}^{l}=\text{G}(X_{t}^{l},W_{t}^{l})=(1-Z_{t}^{l}{)}^{\circ }W_{t-1}^{l}+{Z_{t}^{l}}^{\circ }{\hat{W}}_{t}^{l} \end{array}$$ (2)

$$Z_{t}^{l}=\text{sigmoid}(U_{z}^{l}X_{t}^{l}+V_{z}^{l}W_{t-1}^{l}+B_{z}^{l})$$ (3)

$$R_{t}^{l}=\text{sigmoid}(U_{R}^{l}X_{t}^{l}+V_{R}^{l}W_{t-1}^{l}+B_{R}^{l})$$ (4)

$${\tilde{W}}_{t}^{i}=\text{tanh}(U_{W}^{i}X_{t}^{i}+V_{W}^{i}({R_{t}^{i}}^{\circ }W_{t-1}^{i})+B_{W}^{i}$$ (5)

where $Z_{t}^{l}$, $R_{t}^{l}$, and ${\tilde{W}}_{t}^{l}$ are the update gate output, the reset gate output, and the pre-output, respectively. The update of the weight matrix can be viewed as applying the standard GRU operation to each column of the matrix. While the standard GRU operation is for between vectors, the process of updating the GCN weight matrix is for between matrices. The weight matrix $W_{t}^{l}$ at moment *t* is used as the hidden state of the GRU; the node representation matrix $X_{t}^{l}$ of layer l at moment t is used as the input of the GRU unit *t*o introduce the information of the current moment; the GRU unit outputs the updated $W_{t+1}^{l}$, which is used as the weight matrix of the next moment. The calculation of $W_{t+1}^{l}$ includes both historical and current moments. Since the weight matrix $W_{t}^{l}$ has different column dimensions from the node representation matrix$X_{t}^{l}$, a new sampling of $X_{t}^{l}$ is added to the network layer operations in this layer to achieve the same number of columns as B. The GCN module aggregates the node’s neighborhood information from bottom up, while the GRU module updates the weight parameters from left to right over the time dimension. As a result, the spatial convolutional layer dynamically obtains the neighborhood information of a node. The operation of the spatial convolutional layer can be formalized as follows:


$$\begin{array}{c} X_{t}^{l+1}=\text{F}(X_{t}^{l},A_{t},W_{t}^{l})=\text{F}(X_{t}^{l},A_{t},\text{G}(X_{t}^{l},W_{t}^{l})) \end{array}$$ (6)

where the functions $\begin{array}{c} F \end{array}$ and $\begin{array}{c} G \end{array}$ denote the graph convolution operation and weight update operation, respectively.

### 3.4 Time convolution layer

**Fig 4:**
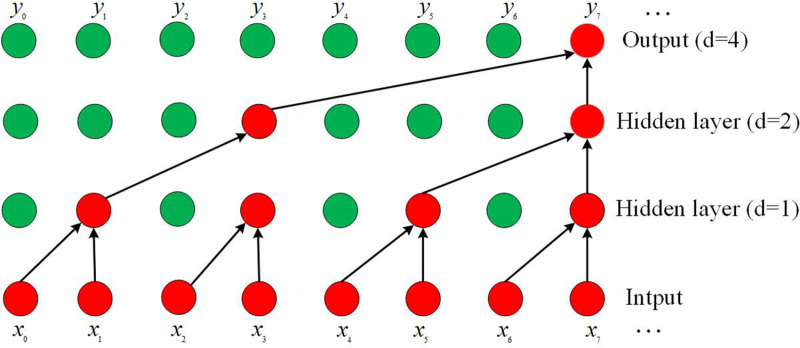
Dilated convolution process in TCNs.

Extracting timing information in dynamic graph node representation learning is a very critical part. Most of the existing models use RNN architecture to model timing changes, which is more time-consuming and memory-consuming due to the complex gate mechanism of RNN-based architecture. Meanwhile, the standard RNN is prone to the problem of gradient disappearance during training, and can only acquire short-term memory, and cannot handle long-time sequences well. Although LSTM and GRU solve the limitations of gradient vanishing and short-term memory to a certain extent, they are still at a disadvantage in terms of computing speed, memory consumption, and performance performance compared to the temporal convolutional neural networks (TCN) - based architecture, so our model adopts the TCN -based architecture to obtain historical information [26]. Compared with the RNN architecture, the TCN can greatly improve the speed of computation due to the parallelizability of convolutional operations, and its computation also consumes less memory. Importantly, the TCN structure is more flexible with respect to the sensory domain of historical information, and the sensory domain can be increased by simply enlarging the convolution kernel or increasing the dilation scale of the dilation convolution in order to obtain information for longer time series. On the other hand, the RNN architecture models the dynamics by simply summarizing the historical information into the hidden states at each moment, and memorizing the historical information through the hidden states [27]. TCN’s convolutional structure, on the other hand, combines the historical information with the current moment’s information through the flexibility of information aggregation, so as to extract the temporal and structural information in the dynamical graphs, and also unify the temporal convolution with the spatial convolution in another way. The temporal convolution and spatial convolution are also unified from another perspective. The temporal convolution layer consists of a one-dimensional fully - connected convolution module and a causal convolution module. The one-dimensional fully - connected convolution operation ensures that the output layer has the same sequence length as the input layer, while the causal convolution operation ensures that the outputs at moment *t* are only convolved with the moments prior to it, thus guaranteeing that the prediction of future moments is modelled from the current moment and the historical information [28]. In the causal convolution stage, in order to make the network structure for longer time series also have a longer and more flexible sense domain, in the causal convolution to join the expansion of the convolution set, with the increase in the number of network layers, the expansion of the convolution is set to the number of layers of the power of two. Fig 4 shows the expansion convolution process in TCN, where the expansion size of each layer is 1, 2, and 4 in turn, and the convolution kernel size is 2. In Fig 4, the green circle represents the convolution kernel used in dilation convolution. In dilation convolution, there are gaps between elements in the convolution kernel, and the size of these gaps is determined by the dilation factor. Convolution kernels with different dilation factors are illustrated in Fig 4. The red circles represent the hidden states in the network. Each hidden state is computed by applying the convolution kernel to the hidden state of the input sequence or the previous layer. Due to the spacing between elements in the convolution kernel, each hidden state in dilation convolution can capture a broader range of input information.

Formally, given an input sequence $X^{l}\in R^{T\times M}$, with M -dimensional features, of length T in layer *l*, and a filter f:{0,1,…,k−2}, the convo*l*ution operation is defined for an element x of $X^{l}$ as follows:


$$ℋ(x)=(X^{l}{*}_{d}\;f)(x)=\sum_{i=0}^{k-2}f(i)\cdot X_{x-d\cdot i}^{l}$$ (7)

Where *d* is the dilation factor, *k* is the filter size, and x−d·i represents the direction of the history. We can increase the sensory domain of convolution by increasing the filter size *k* or increasing the expansion factor d. At the same time, the convolution operation supports the parallel operation on the sequence, so there is a great improvement in the efficiency. The node embedding vectors after spatial convolution in the *l*th layer of the spatia*l* convolution model are the aggregation of the neighbour information, and after the temporal convolution layer, we can get the historical information of each node and the node neighbours. By superimposing a spatial convolutional layer on top of this, the vector representation of each node contains an aggregation of information about the current and historical moments of it and its neighbors. Therefore, this model structure of temporal convolution and spatial convolution overlapping each other makes the output node embedding vectors have richer structural and historical information.

### 3.5 Model training

In order to test the representation ability of the model, we train the model in a specific edge classification task. The edge classification task is highly relevant in many real-world scenarios, such as the identification of crimes in a financial network, which requires the study of edge classification on the connecting edges between two accounts. The task of edge classification under dynamic graphs aims at predicting the edge labeling category of an edge (*u*,*v*) at the moment *t*. In order to classify an edge, we need the node vector representations of the two endpoints of the edge. Given that the vector representations of two nodes *u* and *v* connected by an edge at moment *t* are$X_{t}^{u}$ and$X_{t}^{v}$, the parameter matrix P is used to predict the label of the edge (*u*,*v*):


$$y_{t}^{u\nu }=\text{softmax}(P[X_{t}^{u};X_{t}^{\nu }])$$ (8)

$$L=-\sum_{t=1}^{T}\sum_{\left(u,v\right)}\alpha_{uv}\sum_{i=1}^{N}(z_{t}^{uv}{)}_{i}\log(y_{t}^{uv}{)}_{i}$$ (9)

The cross-entropy loss function of the model is:

where$z_{t}^{uv}$ denotes the true labeling categories of the edges; the weight parameter$\alpha_{uv}$ is a hyperparameter that balances the weights of the category distributions, and the experimental datasets all suffer from severe category imbalance, and the weights of the categorized categories are balanced by adjusting$\alpha_{uv}$. The weight parameter$\alpha_{uv}$ is a hyperparameter that balances the weights of the categorized categories.

## 4 Results and discussion

This study used BIM technology to obtain a data set, and used DynGCN to forecast the cost of power engineering. Based on BIM technology platform, visual models for various engineering schemes can be built, and each scheme has its corresponding cost prediction model. By associating the BIM model with the cost estimate, the cost estimate data set of each scheme is generated. The obtained data set is divided into two parts: training data and test data. The training data is simply standardized, and the pre-processing operations such as data denoising and normalization are completed. In order to avoid the interference caused by bad data in training data, batch processing mode is used for network training; that is, a certain number of training samples are randomly selected to form a small sample as input. By updating the weight value of each small sample until the iteration stop condition is satisfied, the network training is completed. Then the test data of power engineering cost is input into the trained DynGCN network, and the predictive optimization value of cost is obtained by using forward propagation.

Different types of electric power engineering projects are influenced by different proportions of factors. The model in this study may not be fully applicable to all types of electric power engineering projects, especially in terms of the unique cost factors of specific types of projects. For example, in thermal power construction projects, with the improvement of environmental standards, more funds need to be invested in the construction and operation of environmental protection facilities such as desulfurization, denitrification, and dust removal. This requires the model in this study to place more emphasis on this factor when predicting the cost of thermal power construction projects, which may increase the complexity of the model, requiring more complex computational resources and longer training times.

In order to verify the reliability of the proposed algorithm and the optimization performance of the project, the relevant engineering cost data of a provincial power company are collected. These data cover different types of projects, including but not limited to equipment procurement, engineering construction, and maintenance. 400 of these items were selected as sample data for this study. The sample data covers a wide range of project categories and different project sizes to ensure that the validation results are widely applicable and reliable, and some of the data are shown in Table 1. The first 326 data items were used as the training set and the last 74 data items were used as the test set.

**Table 1:** Part of the investment data of a power project.

Project type	Project specific item	Investment amount/ Wan Yuan
Accessory engineering	Transformer equipment	845.08
	Power distribution equipment	1000.55
	Monitoring microcomputer system	329.29
	⁝	⁝
Foundation engineering	Station area occupancy	248.14
	Electrical distribution room	261.96
	⁝	⁝
Erection engineering	–	2513.72
Pole and tower engineering	–	1285.01
Total investment	–	9669.83

Meanwhile, predictive models are built based on the Pytorch deep learning framework for analyzing the selected power engineering datasets. In addition, the optimization performance of the proposed algorithm is evaluated in the experiments using the cost level prediction accuracy $P_{L}$, which is calculated as the expression.


$$P_{L}=[1-\sqrt{\frac{1}{\text{n}}{\sum_{i=1}^{n}\left(\frac{\hat{Y}-Y}{Y}\right)}^{2}}times100\%$$ (10)

### 4.1 Methodological accuracy

**Fig 5:**
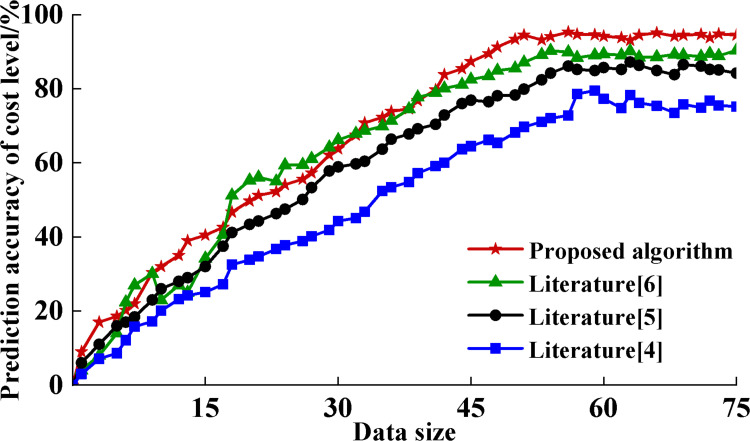
Comparison results of forecast accuracy of consolidated cost levels.

To further validate the performance of the algorithm, this study analyzes the proposed algorithm in comparison with the results of other existing studies. This comparison aims to assess the relative performance of the proposed algorithms in the prediction of integrated cost levels. The model weights are initialized using a normal distribution, and the Adam optimizer is employed. The dropout rate is set to 0.3, and the learning rate is 0.001. During training, cross-entropy is used as the loss function, with an L2 regularization term added, and the weight of L2 regularization is set to 0.0005. The model is trained for a total of 50 epochs. Additionally, the input data are standardized in this study. Fig 5 shows the detailed data of different algorithms in terms of prediction accuracy.

From the data in Fig 5, it can be observed that all the algorithms show some improvement in prediction accuracy as the amount of data increases. However, while all algorithms achieved better prediction results when the amount of data increased, our proposed algorithm performed particularly well, with a prediction accuracy of nearly 96%, significantly higher than the other compared algorithms. This performance improvement is attributed to the following areas:

(1) Firstly, the proposed algorithm incorporates BIM technology in engineering cost prediction. BIM technology provides detailed 3D models and rich life cycle information of power engineering projects, enabling accurate digital management of each stage from design to construction. This comprehensive life-cycle perspective allows the algorithm to collect and utilize more engineering data at different stages, which in turn improves the accuracy of the predictions [29].
(2) Secondly, the proposed algorithm also utilizes the DynGCN technique. DynGCN is capable of dynamically processing and updating engineering data for each period, capturing temporal and spatial relationships in the data through a complex graph neural network structure. This dynamic processing capability allows the algorithm to adjust and optimize the prediction at each stage in real time, thus improving the prediction performance in general [30].

In contrast, although the algorithms proposed in literature [4] and literature [5] are also based on BIM technology, their implementations are only focused on some specific phases of engineering cost management and lack a comprehensive optimization of the entire project life cycle. This approach may fail to fully utilize data from all phases of the project, leading to limitations in its predictive performance. In addition, although the MK-TESM method in the literature [6] is also used for engineering cost estimation, the method mainly focuses on some specific cost estimation techniques and lacks global control over the whole life cycle of power projects. This limitation makes it difficult for the MK-TESM method to exceed 90% prediction accuracy when dealing with complex and dynamic power engineering projects.

**Fig 6:**
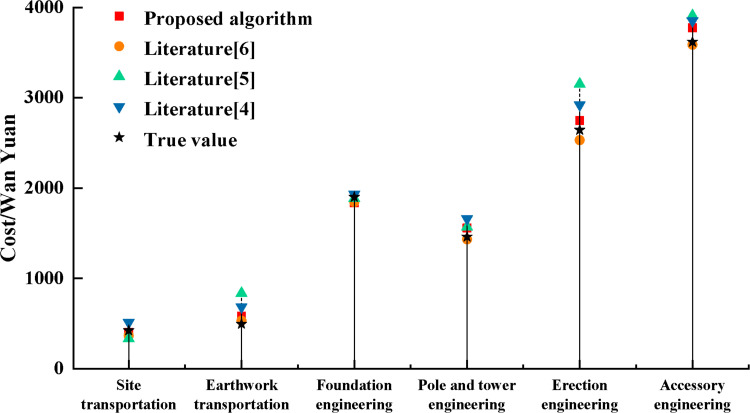
Comparison of cost results by project.

In order to demonstrate the cost optimization effect of the proposed algorithm on each engineering node, the predicted cost was compared and analyzed with literature [4,5] and [6] in various engineering aspects, and the results are shown in Fig 6.

As can be seen in Fig 6, the algorithm proposed in this study has a relatively high degree of agreement with the actual values in predicting the cost of most of the projects. Specifically, the algorithm demonstrates superior predictive ability in most projects, effectively approximating the true engineering cost. However, in the prediction of infrastructure projects, its performance is slightly inferior to the algorithms proposed in the literature [6]. This difference stems from the specificity and complexity of infrastructure projects, which require additional adjustments and optimization. The main innovation of the algorithm proposed in this study is the integration of BIM technology into the whole process control of power engineering. BIM technology provides an integrated, visual way to manage project information, enabling precise control and optimization at every stage, from design to construction. Based on this, this study used DynGCN for cost prediction. The introduction of DynGCN enables the dynamic processing and updating of engineering data to reflect the actual situation of the project in real time, thus providing higher accuracy in the forecasting process. By optimizing engineering decisions and timely adjusting construction plans, prediction bias can be significantly reduced, making cost prediction more accurate and reliable [31]. In contrast, other comparative algorithms are analyzed only at the level of engineering data or a single algorithm. These algorithms focus only on a particular data feature or computational method and fail to fully integrate all aspects of the engineering project, resulting in their overall poor performance. In addition, the algorithm proposed in this study not only considers the static data of the project, but also integrates the real-time dynamic data, which enables the prediction model to adapt to the changes in the progress of the project in a timely manner. This comprehensive and dynamic treatment, which is lacking in traditional algorithms, greatly improves the adaptability and accuracy of engineering cost forecasting.

### 4.2 Computational complexity

The computational complexity of an algorithm measures the time and space resources required by the algorithm to process the input data. The smaller the computational complexity, the less time the algorithm takes to run and test, and the faster it predicts the cost of the project [32]. Therefore, this study evaluates the complexity of the DynGCN algorithm, as well as the methods proposed in literature [4], literature [5], and literature [6]. The computer system used in this study was Windows 10 64-bit with an Inter(R) Core i5-1155G7 CPU and 16.0 GB RAM. The programming language is Python 3.7, using the PyTorch open source framework. The running time and testing time required for different methods to achieve the same accuracy are shown in Table 2.

**Table 2:** Running time and testing time required for different methods for engineering cost prediction.

Algorithms	DynGCN	Literature [4]	Literature [5]	Literature [6]
Running time (s)	12.56	25.43	20.31	16.56
Testing time (ms)	78	140	120	87

As can be seen from Table 2, the DynGCN algorithm proposed in this study consumes the least amount of runtime and testing time for engineering cost prediction compared to the methods proposed in literature [4], literature [5], and literature [6]. This shows that the DynGCN algorithm not only performs well in terms of computational complexity, but also effectively reduces the time cost in the prediction process. This efficient computational performance makes the DynGCN algorithm have significant practical applications in power engineering cost prediction. It can quickly and accurately complete the cost prediction task and provide timely support for engineering decision-making.

### 4.3 Ablation experiment

The dynamic graph update mechanism is a key component of DynGCN, which dynamically adjusts the graph structure based on changes in the data and the graph’s state (e.g., node and edge characteristics) during the training process. This mechanism significantly improves the model’s ability to adapt to structural changes in the graph and helps it better capture the complex, dynamic relationships between nodes. Therefore, in the ablation experiments, the impact of removing this updating mechanism on the model’s performance is explored by comparing a version of the model with and without this mechanism. The performance of both models is evaluated based on the prediction accuracy of the integrated cost level, and the results are shown in Table 3.

**Table 3:** Influence of dynamic graph update mechanism on prediction accuracy of integrated cost level.

Data size	15	30	45	60	75
Prediction accuracy (Dynamic graph update mechanism)	40.72%	62.64%	87.32%	94.12%	95.98%
Prediction accuracy (No dynamic graph update mechanism)	35.78%	54.53%	79.21%	85.45%	89.23%

As shown in Table 3, the prediction accuracy improves as the dataset size increases, regardless of whether the dynamic graph updating mechanism is used. However, across all dataset sizes, the model with the dynamic graph updating mechanism consistently outperforms the model without it in terms of prediction accuracy. This indicates that the dynamic graph updating mechanism is effective in enhancing the model’s prediction accuracy at all dataset scales.

### 4.4 Scalability of the method

The cost of different types of power engineering is influenced by various factors, which include but are not limited to weather conditions, geographical location, etc. From the outset of designing this model, this key point was taken into consideration, and the model has the capability to incorporate new variable factors. Without altering the core prediction mechanism, this model can easily integrate new input features, thus adapting to the evaluation needs of different power cost sectors. When this algorithm is applied to other power cost sectors, the new variable factors can be integrated into the existing variable framework of the original model. This may lead to an increase in model complexity and the workload of data processing, but it will not affect the final evaluation results. Therefore, the algorithm has a certain degree of scalability for such applications. However, if a large number of additional variables are introduced, it may change the structure of the model itself, and the training time of the model may significantly extend. Additionally, the consumption of computational resources will increase, and ultimately, it may lead to inaccurate cost evaluation results, meaning the model may no longer be well-matched. Although this algorithm has a certain degree of scalability, when applied to very complex environments, it is necessary to make corresponding adjustments and optimizations to address the impact of the new variables to ensure the predictive performance and stability of the model.

## 5 Conclusion

In the past, power engineering cost estimation mainly relied on manual analysis and speculation by experienced personnel. This method not only relies on personnel expertise and experience, but also has a high error rate and is difficult to dynamically adjust in real time. To solve these problems, this study combines BIM technology and DynGCN to propose a new method for cost prediction of electric power projects. BIM technology can realize the dynamic control of the whole life cycle of power engineering, and can track and manage all the data in the design, construction and operation and maintenance processes of power engineering in real time. DynGCN is able to efficiently handle data with spatial and temporal dimensions. Compared to traditional CNNs, DynGCN is not only capable of handling spatial features, but also combines time series information to provide a more comprehensive and deeper feature representation. Compared with traditional recurrent RNNs and LSTMs, DynGCN performs better in handling long-term dependencies and complex temporal variations. In this study, the cost optimization algorithm for electric power engineering based on BIM and DynGCN not only improves the accuracy and real-time cost estimation, but also optimizes the cost management scheme of the whole project, thus ensuring both the quality of the engineering and the economy. In future research, the following steps will be taken: Firstly, for power engineering projects of different scales and types, we will analyze the characteristics of each project type and develop corresponding model design criteria. Through experimental validation and adjustment of model parameters, we aim to tailor the model to meet the specific needs of different projects, thereby designing more targeted model structures and parameter optimization strategies. Secondly, we will enhance data preprocessing efforts to improve the quality and completeness of the data, with the goal of increasing the predictive accuracy of the model. Finally, we will construct a training dataset that spans different project types, enabling the model to learn the general features of various project types. We will conduct model training and validation across multiple project types to ensure the model’s predictive accuracy in different scenarios, thereby improving the model’s generalization capabilities.
